# From Surface Energetics to Environmental Functionality: Mechanistic Insights into Hg(II) Removal by L-Cysteine-Modified Silica Gel

**DOI:** 10.3390/gels12020141

**Published:** 2026-02-03

**Authors:** Rene G. Moran-Salazar, Ricardo Manríquez-González, Alejandro A. Peregrina-Lucano, José A. Gutierréz-Ortega, Agustín Lara, Eulogio Orozco-Guareño, Adriana M. Macias-Lamas, Jessica Badillo-Camacho, Ilya G. Shenderovich, Milton Vazquez-Lepe, Sergio Gómez-Salazar

**Affiliations:** 1 Department of Chemistry, University Center of Exact Sciences and Engineering, Universidad de Guadalajara, Blvd. Marcelino García Barragán #1421, esq. Calz. Olímpica, Guadalajara 44430, Mexico; rene.moran@academicos.udg.mx (R.G.M.-S.); lafe@cucei.udg.mx (A.L.); eulogio.orozco@academicos.udg.mx (E.O.-G.); 2 Department of Wood, Cellulose and Paper, University Center of Exact Sciences and Engineering, Universidad de Guadalajara, Km 15.5, Carretera Guadalajara-Nogales, Zapopan 45020, Mexico; ricardo.manriquez@academicos.udg.mx (R.M.-G.); joseantonio.gutierrez@academicos.udg.mx (J.A.G.-O.); jessica.bcamacho@academicos.udg.mx (J.B.-C.); 3 Department of Pharmacobiology, University Center of Exact Sciences and Engineering, Universidad de Guadalajara, Blvd. Marcelino García Barragán #1421, esq. Calz. Olímpica, Guadalajara 44430, Mexico; aaron.peregrina@academicos.udg.mx (A.A.P.-L.); macaria.macias@academicos.udg.mx (A.M.M.-L.); 4 Faculty of Chemistry and Pharmacy, University of Regensburg, Universitaetstrasse 31, 93053 Regensburg, Germany; ilya.shenderovich@chemie.uni-regensburg.de; 5 Departamento de Ingeniería de Proyectos, University Center of Exact Sciences and Engineering, Universidad de Guadalajara, José Guadalupe Zuno 48, Belenes Industrial, Zapopan 45100, Mexico; milton.vazquez@academicos.udg.mx; 6 Department of Chemical Engineering, University Center of Exact Sciences and Engineering, Universidad de Guadalajara, Blvd. Marcelino García Barragán #1421, esq. Calz. Olímpica, Guadalajara 44430, Mexico

**Keywords:** mercury remediation, zwitterionic silica adsorbent, L-cysteine functionalization, surface energy distribution (DFT), sustainable water treatment

## Abstract

The development of oxidation-resistant and regenerable materials remains a major challenge for mercury removal from contaminated waters and industrial effluents. In this study, a zwitterionic mesoporous silica gel functionalized with L-cysteine (SG-3PS-Cys) was synthesized, where the thiol group is covalently anchored to the silica framework, preventing oxidative degradation while preserving –NH_3_^+^ and –COO^−^ groups for Hg(II) coordination. Spectroscopic analyses (FTIR, XPS, and ^13^C NMR) confirmed the formation of a stable, thiol-free binding environment in which mercury interacts through carboxylate oxygen atoms, electrostatically stabilized by neighboring ammonium groups. The material exhibited a high surface area (134 m^2^ g^−1^) and uniform mesoporosity (9.8 nm), achieving a maximum Hg(II) uptake of 82.7 mg g^−1^ at pH 3 with rapid kinetics and cooperative S-type isotherms. The adsorbent retained 72% of its capacity after five regeneration cycles and maintained 38.7% selectivity toward Hg(II) in multicomponent solutions. DFT-based surface energy distribution analysis supported the zwitterionic coordination mechanism, revealing energetically homogeneous and high-affinity binding domains. Beyond its chemical stability, the material introduces a sustainable route for mercury remediation, linking surface energy, electrostatic effects, and porosity to achieve durable performance under acidic and complex aqueous conditions. These findings provide a mechanistic and design framework for the next generation of non-thiol adsorbents capable of selective and reusable Hg(II) removal in environmentally relevant scenarios.

## 1. Introduction

Mercury (Hg) contamination remains a critical global environmental concern due to its extreme toxicity, persistence, and bioaccumulation in aquatic ecosystems [1]. Industrial activities such as chlor-alkali production [2], mining [3] and waste incineration [4] release significant amounts of Hg(II) into wastewater, threatening both ecological and human health. According to the World Health Organization [5] and the United Nations Environment Programme (UNEP) [6], the permissible concentration of total mercury in drinking water is ≤1 µg L^−1^, emphasizing the urgent need for efficient and regenerable sorbents capable of removing trace Hg(II) levels under realistic conditions. Conventional treatment methods such as precipitation, ion exchange, or membrane filtration often suffer from high operational costs or poor selectivity, driving research toward adsorption-based technologies that combine chemical selectivity with scalability [7,8,9].

Recent advances have focused on functionalized mesoporous silicas and hybrid organic–inorganic materials due to their large surface area, tunable pore architecture, and versatile surface chemistry [7,10,11]. Two major strategies dominate mercury sorbent design: sulfur-based and non-sulfur (zwitterionic) functionalization. The former employs thiol, thioether, or thiocarbonyl groups as coordination centers, exploiting the classical soft–soft interaction between Hg(II) and sulfur. However, such materials often exhibit oxidative degradation and limited recyclability under acidic or oxidizing environments, reducing their long-term applicability [12]. In contrast, the non-sulfur or zwitterionic route uses oxygen and nitrogen donor groups such as –COO^−^ and –NH_2_, enabling Hg–O and Hg–N coordination stabilized by adjacent –NH_3_^+^ moieties [13]. This second strategy enhances oxidative stability, sustainability, and reusability—core priorities in environmental technology [10,14,15].

In addition to conventional textural parameters, the surface–energy distribution derived from N_2_ adsorption data using density functional theory (DFT) provides a powerful means to describe the energetic heterogeneity of porous adsorbents [16]. DFT analysis translates adsorption isotherms into adsorption–energy spectra that quantify the population of sites with different binding strengths. Low-energy domains (ε/k ≈ 20–40 K) correspond to weak physisorption on siloxane walls, whereas high-energy domains (ε/k > 80–100 K) are associated with functionalized or defect-rich regions capable of stronger binding and greater stability during regeneration cycles [17]. Such energetic mapping has been successfully applied to mesoporous silicas, carbons, and hybrid adsorbents to explain the relationship between surface chemistry, pore topology, and adsorption efficiency [7,8,18,19]. In this work, DFT-derived surface–energy distributions are used to correlate the presence of zwitterionic –COO^−^/–NH_3_^+^ domains introduced by L-cysteine functionalization with the emergence of high-energy adsorption sites responsible for Hg (II) uptake and material reusability. This analysis bridges surface energetics and environmental performance, offering a mechanistic basis for designing durable and regenerable adsorbents for sustainable water treatment.

Among amino-acid-derived materials, L-cysteine (Cys) offers exceptional versatility due to its multifunctional donor sites (–COOH, –NH_2_, and –SH). Cys-based silicas have been extensively studied for Hg(II) removal, owing to their strong Hg–S affinity [11,20,21]. Nonetheless, thiol oxidation and disulfide formation significantly compromise their regeneration efficiency. The present study introduces a distinct approach in which the sulfur atom of L-cysteine is covalently immobilized within the silica network (Si–C–S– linkage), leaving the amino and carboxylate groups available for metal coordination. This configuration produces a thiol-free, zwitterionic surface capable of binding Hg(II) through cooperative –COO^−^/–NH_3_^+^ interactions rather than direct Hg–S bonding. Such a design ensures enhanced durability and chemical resistance under acidic or oxidative conditions, aligning with sustainable wastewater remediation principles [7,9,14].

The novelty of this work lies in demonstrating a non-thiol Hg(II) coordination pathway on L-cysteine-functionalized silica, confirmed by FTIR, ^13^C NMR, and XPS, representing one of the first studies to validate zwitterionic adsorption as a sustainable alternative to sulfur-dependent mechanisms. By integrating chemical durability, high selectivity, and multi-cycle regeneration, the developed adsorbent addresses key challenges for industrial water treatment namely, stability in acidic effluents, reusability, and low environmental impact. This approach offers a new route toward mercury remediation: durable, recyclable, and non-toxic adsorbents rooted in amino acid chemistry rather than traditional thiol coordination.

The objectives of this study were as follows: (i) to synthesize a mesoporous silica functionalized with L-cysteine via a propyl linker where sulfur is covalently anchored (Si–C–S–); (ii) to evaluate adsorption capacity, kinetics, multi-ion selectivity, and regeneration efficiency for Hg(II) removal; (iii) to elucidate the adsorption mechanism using FTIR, solid-state NMR, and XPS; and (iv) to correlate surface–energy distribution (from DFT modeling) with zwitterionic functional-group energetics. These aims collectively demonstrate how controlling interfacial chemistry at the molecular scale can yield environmentally robust sorbents for heavy-metal remediation.

## 2. Results and Discussion

### 2.1. FTIR Analysis of the Functional Precursor 3PTES-Cys

**Figure 1:**
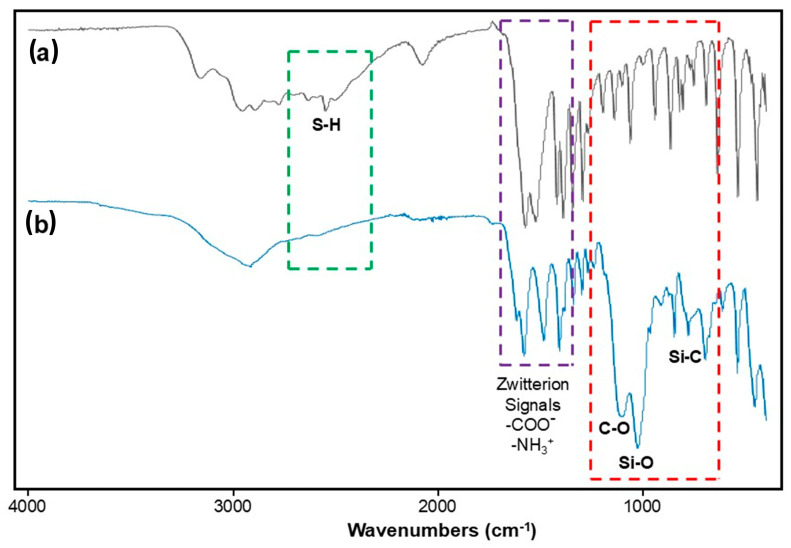
FTIR spectra of (a) cysteine and (b) functional precursor 3PTES-Cys. The dashed rectangles indicate the main signal changes between both spectra associated with the differences in the chemical composition of cysteine before and after 3PTES.

Figure 1 presents the FTIR spectra of cysteine (a) and the functional precursor 3PTES-Cys (b). The main spectral changes associated with the covalent bonding between cysteine and the propyl-silane connector (3PTES) are highlighted in three frequency regions. (i) Green dashed region: the characteristic S–H stretching band of cysteine at 2551 cm^−1^ (Figure 1, (a)) disappears after functionalization (Figure 1, (b)), confirming the formation of a thioether bond between the cysteine sulfur and the propyl-silane group [22,23]. (ii) Purple dashed region (1700–1400 cm^−1^): these bending and stretching vibration bands correspond to the zwitterionic groups (–COO^−^ and –NH_3_^+^) of cysteine [24,25]. Noticeable shifts and partial splitting are observed when comparing spectra, a and b, indicating changes in the local environment and association between the amino and carboxylate moieties upon 3PTES bonding [22,24]. (iii) Red dashed region: new absorption bands at 1110, 1026, and 800 cm^−1^ appear in the 3PTES-Cys spectrum, assigned, respectively, to C–O, Si–O, and Si–C stretching vibrations of the ethoxysilane network formed during functionalization. These bands are absent in pristine cysteine (Figure 1, (a)), confirming successful grafting of the 3PTES moiety. Overall, these spectral modifications confirm that cysteine successfully reacted through its thiol group to form the 3PTES-Cys precursor, while the amino and carboxylate groups remained available for subsequent coordination reactions.

### 2.2. Hg(II) Adsorption Kinetics on SG-3PS-Cys

**Figure 2:**
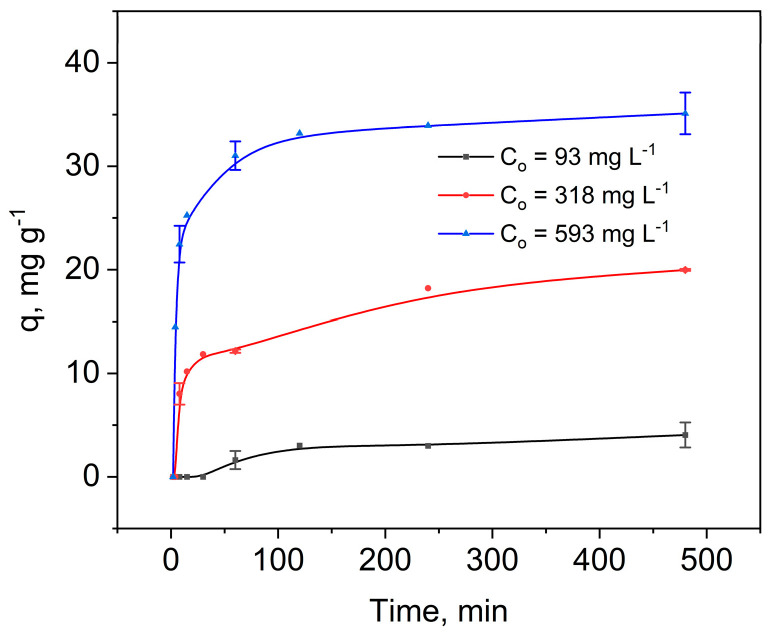
Adsorption kinetics of Hg(II) on SG-3PS-Cys at three initial concentrations (C_0_ = 93, 318, 593 mg L^−1^) in 0.1 M acetate buffer (pH 3, 298 K). 0.2 g adsorbent/20 mL solution. Symbols: experimental data; lines: visual guides.

The adsorption kinetics of Hg(II) ions on SG-3PS-Cys were evaluated to determine the equilibrium time and rate behavior. Three initial concentrations (93, 318, and 593 mg L^−1^) were tested to assess the effect of concentration on adsorption rate (Figure 2). The uptake capacity increased proportionally with C_0_, consistent with greater occupation of binding sites at higher driving force [26]. The apparent plateau (~38 mg g^−1^ at 593 mg L^−1^) corresponds to kinetic rather than equilibrium conditions (0.2 g adsorbent, 25 °C, 120 min). At the lowest concentration (93 mg L^−1^), a short induction period (~25–30 min) preceded measurable adsorption, attributed to a weaker concentration gradient, the lower driving force for mass transfer, which slows down the initial adsorption stage and partial protonation of surface groups at pH 3, which temporarily limits coordination until electrostatic rearrangement occurs. Once adsorption starts, the rate accelerates and equilibrium is reached in ≈120 min across all concentrations, indicating a common rate-controlling mechanism. The true equilibrium capacity (82.7 mg g^−1^, pH 3) was later obtained from isotherm data (Section 2.3). Although these concentrations exceed environmental levels, they were selected to ensure quantifiable kinetics. Equilibrium was reached at ≈120 min, after which q remained constant up to 480 min, confirming rapid kinetics.

**Figure 3:**
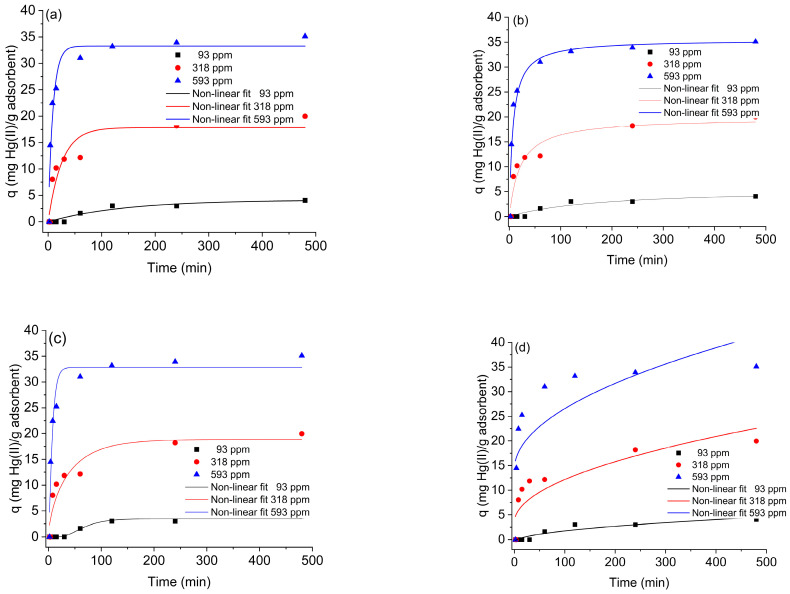
Kinetic model fittings for Hg(II) adsorption on SG-3PS-Cys (pH 3, 298 K, acetate 0.1 M, 0.2 g/20 mL). (**a**) PFO; (**b**) PSO; (**c**) Avrami; (**d**) Intraparticle diffusion. Symbols: experimental data; lines: nonlinear fits.

To identify the rate-limiting step, four models were tested: pseudo-first-order (PFO), pseudo-second order (PSO), intraparticle diffusion (ID), and Avrami (Figure 3a–d). The fitting parameters are summarized in Table 1. The PFO model showed poor agreement (R^2^ = 0.882 at 318 mg L^−1^, qₘₐₓ two units below the experimental value). The ID model also underperformed (R^2^ < 0.9 for 318 and 593 mg L^−1^). Although the Weber–Morris plot is often divided into several linear regions, recent studies have demonstrated that such segmentation is arbitrary and lacks physical basis [27]. For this reason, a single continuous regression of q = A t^1/2^ + B was preferred. Among all models, the PSO equation yielded the best description (R^2^ = 0.919–0.927; qₘₐₓ in excellent agreement with experiment) and the lowest ΔAIC values, confirming its superior fit. The Avrami model also performed reasonably well at 93 and 593 mg L^−1^ but deviated at 318 mg L^−1^, indicating limited applicability. PFO and ID models showed ΔAIC > 10, thus statistically unsupported. Figure 3b illustrates the PSO fittings, which reflect a second-order dependence on the number of active sites, implying that each Hg(II) ion interacts with two functional groups on the surface [28,29]. Such behavior is characteristic of chemisorption involving ion-exchange or complexation steps controlling the overall rate.

### 2.3. Equilibrium Adsorption Isotherms

**Figure 4:**
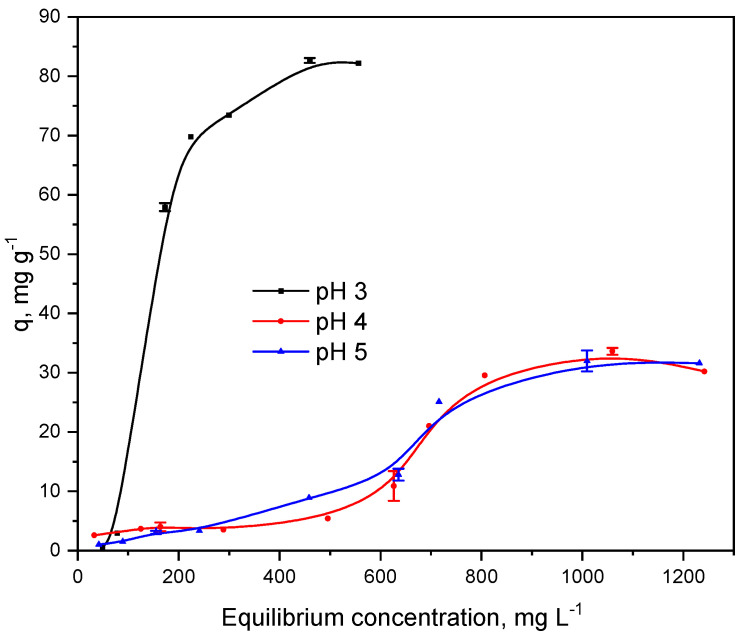
Adsorption isotherms of Hg(II) ions on the SG-3PS-Cys adsorbent at three pH values. Symbols represent the mean of triplicate measurements (±1 SD); continuous lines are visual guides.

Adsorption isotherms were obtained using Hg(II) solutions with initial concentrations of 50–1500 mg L^−1^ at pH 3, 4, and 5 values representative of industrial wastewater [30]. As shown in Figure 4, Hg(II) uptake begins effectively at pH 3, with equilibrium at ~376 mg L^−1^ and a maximum capacity of 82.7 mg g^−1^ at 460 mg L^−1^. According to aqueous-speciation modeling (pH 3, I = 0.1 M, acetate buffer) and the NIST stability constants database [31], Hg(II) occurs mainly as the anionic complex Hg(Ac)_4_^2−^. Under these conditions, the SG-3PS-Cys surface bearing protonated –NH_3_^+^ and partially deprotonated –COOH groups interacts electrostatically with these anionic species. This interpretation, consistent with FTIR, XPS, and ^13^C NMR analyses, indicates Hg–O coordination rather than Hg–S bonding. While isotherm data alone cannot identify aqueous species, this assignment is supported by combined speciation modeling and spectroscopic evidence.

Following the classification of Giles et al. [32], the obtained curves correspond to S-type isotherms, characterized by an increasing slope with equilibrium concentration typical of cooperative adsorption, where surface coverage enhances further adsorption. The data fits the S-2 subclass, which predicts surface saturation at high loading, reflected by the final plateau. As reported by Giles et al. [32], the initial curvature indicates that adsorption becomes progressively favored as more molecules occupy the surface.

**Figure 5:**
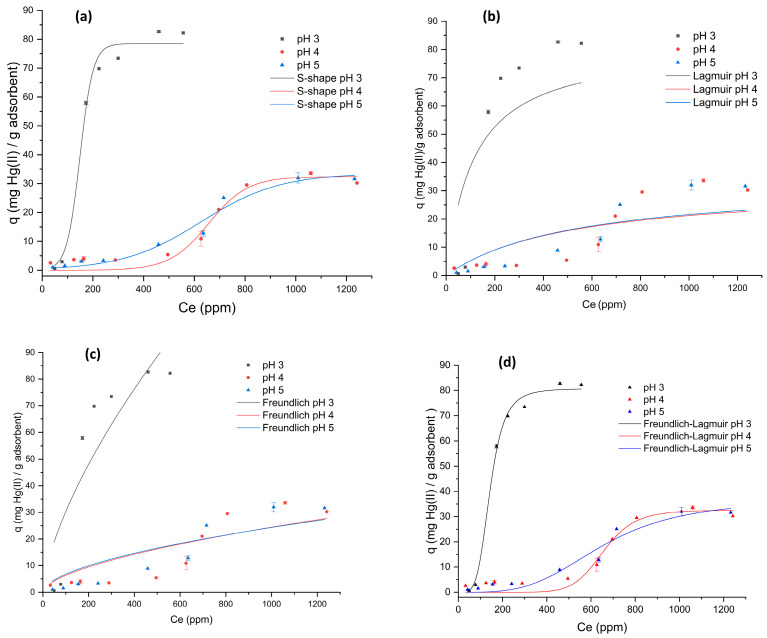
Nonlinear fitting of Hg(II) adsorption isotherms on the SG-3PS-Cys adsorbent using the following models: (**a**) S-shape, (**b**) Langmuir, (**c**) Freundlich, and (**d**) Freundlich–Langmuir. The Langmuir two-site and Toth models are not shown due to negative R^2^ values.

Six nonlinear models, Langmuir, Freundlich, Sips (Freundlich–Langmuir), Langmuir-two-sites, S-shape, and Toth, were tested to describe the adsorption data (Figure 5, Table 2). Although Langmuir and Freundlich cannot reproduce S-type behavior, they were included for comparison and statistical validation across pH values. Among them, only the Sips and S-shape models were physically meaningful because both can account for cooperative effects. Model selection was based on fit quality (R^2^) and physical relevance. The Sips model best described the data at pH 3, while the S-shape model was more suitable for pH 4–5. Despite the limited data at pH 3, the correlations were excellent and the residuals random, confirming model adequacy. At pH 3, the isotherm exhibits a mild S-type profile with q_max_ = 82.7 mg g^−1^. The enhanced uptake at this pH is consistent with electrostatic attraction between cationic surface sites and anionic Hg–acetate complexes (e.g., ${HgAc}_{4}^{2-}$), as suggested by aqueous speciation under acetate buffer and corroborated by the spectroscopic evidence discussed below; however, the isotherm data alone do not identify the specific functional groups involved. At higher pH, surface deprotonation and partial Hg(OH)_2_ formation reduce this attraction, decreasing capacity. The observed S-type profile suggests the coexistence of competing and cooperative adsorption sites. At low pH, electrostatic attraction between the surface and anionic mercury–acetate species dominates, while increasing pH reduces this driving force. The cooperative trend likely originates from local rearrangements of surface domains upon partial coverage, a behavior consistent with cooperative adsorption phenomena reported for hybrid silica adsorbents [33]. Accordingly, the Sips model accurately captures adsorption at pH 3, while the S-shape model describes cooperative mechanisms at pH 4–5. Although qₘₐₓ (82.7 mg g^−1^) is moderate, it is consistent with non-thiol silica systems and confirms that Hg(II) uptake proceeds through reversible Hg–O coordination rather than irreversible Hg–S binding. The combination of fast equilibrium (≈120 min) and >70% regeneration efficiency highlights the chemical robustness and environmental applicability of SG-3PS-Cys. This study demonstrates a stable zwitterionic mechanism capable of efficient, reversible Hg(II) capture, prioritizing sustainability over extreme capacity values. The Langmuir and Freundlich models had poor fits (R^2^ = 0.628–0.786). The Freundlich–Langmuir model showed an R^2^ ≈ 1 only at pH 3, but this was lower at pH 4–5 compared with the S-shape model. The Langmuir two-site and Toth models produced negative R^2^ values at some pH levels, thus being statistically invalid.

### 2.4. Regeneration Study and Multi-Cation Analysis

**Figure 6:**
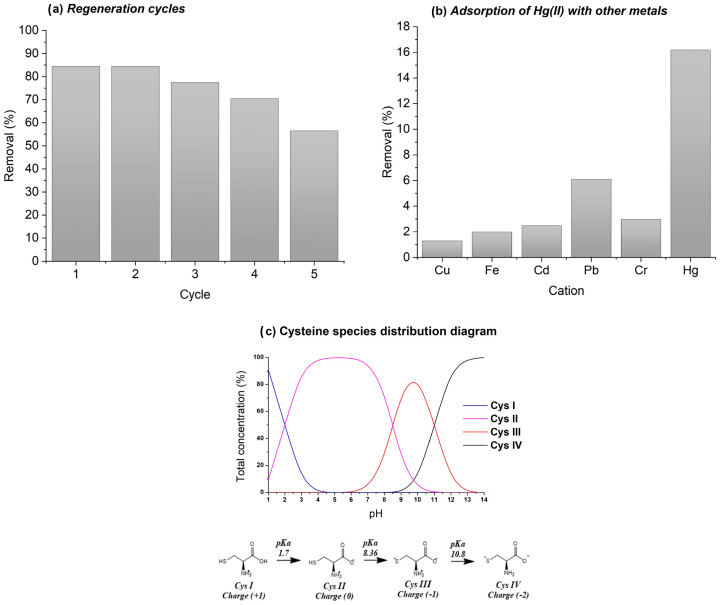
(**a**) Regeneration cycles of Hg(II) adsorption–desorption on SG-3PS-Cys. (**b**) Competitive adsorption of Hg(II) and other metals. In multi-cation tests, Hg was quantified by ICP-MS, while co-ions were measured by AAS to meet detection limits and prevent Hg memory effects. (**c**) Cysteine speciation diagram illustrating the predominant Cys II species at pH ≈ 3.

*Regeneration* (Figure 6a) is key to reducing operational costs and minimizing adsorbent replacement. Five adsorption–desorption cycles were performed using 0.1 M NaOH as regenerant. After four cycles, SG-3PS-Cys retained ~79% Hg(II) removal; in the fifth, efficiency declined by ~28% relative to the first but remained > 70% overall. This performance agrees with non-thiol silica systems, where the absence of –SH groups prevents irreversible Hg–S bond formation, enabling partial desorption and preserving structural integrity. Although thiol-based sorbents often exhibit higher initial capacities, their recyclability is typically lower due to Hg–S bond irreversibility. In contrast, SG-3PS-Cys demonstrates superior stability and reusability under the tested conditions.

*Multi-cation selectivity* (Figure 6b). Because industrial effluents contain multiple metals, competitive adsorption tests were conducted. SG-3PS-Cys showed markedly higher affinity for Hg(II) compared with other cations (Hg ≫ Cr > Fe > Cd > Cu > Pb). This strong selectivity arises from the preferential interaction of Hg(II) with zwitterionic –COO^−^/–NH_3_^+^ surface domains, which provide an electrostatically favorable environment for coordination to oxygen-rich sites. Unlike thiol-based systems governed by soft–soft Hg–S interactions, the selectivity observed here is driven by electrostatic complementarity and the local charge distribution of the zwitterionic interface, promoting stable yet reversible Hg–O coordination. The trend also agrees with the high acetate complexation constant of Hg(II) (log β_4_ ≈ 17) reported in the NIST database [31], indicating that selective uptake results from the synergy between aqueous Hg(Ac)_4_^2−^ species and surface carboxylate sites rather than from sulfur chemistry. The cysteine speciation diagram (Figure 6c) Frank et al. [34] confirms that at pH ≈ 3 the dominant Cys II form carries a protonated amine and a deprotonated carboxylate an optimal configuration for selective Hg(II) binding on the functionalized silica surface. This speciation supports the observed selectivity pattern and explains the high regeneration stability of SG-3PS-Cys, suggesting its potential applicability as a robust and reusable adsorbent for mercury removal from aqueous systems.

### 2.5. Comparative Evaluation of Hg(II) Adsorbents

To contextualize the performance and mechanistic relevance of SG-3PS-Cys, a comparative assessment was made against representative Hg(II) adsorbents from the literature (Table 3). The aminopropyl-functionalized MCM-41 by Hamid et al. [35] reported the highest uptake (125 mg g^−1^, Langmuir model), with outstanding reusability (99.7% retention after 10 cycles) and strong Hg–N coordination. However, the mechanism was not spectroscopically confirmed, and its synthesis involves hydrolysis-prone organosilanes, which may restrict large-scale application. The Cys-C@Fe_3_O_4_ composite of Srikhaow et al. [36] achieved 94.3 mg g^−1^ capacity with partial regeneration over three cycles, relying on thiol and amine coordination. While effective, thiol oxidation limits stability, and magnetite nanocomposites generally require multistep synthesis and show poor acid resistance. Hydride–silica composites reported by Katok et al. [37] reached 101 mg g^−1^ via Si–H surface groups. Yet, their mechanism was inferred rather than demonstrated, and no regeneration data were included. Moreover, hydride groups offer limited selectivity and are susceptible to hydrolysis. The PEI-modified fly ash tobermorite by Liu et al. [38] exhibited moderate capacity (82.6 mg g^−1^) and good regeneration, though its industrial fly ash precursor is compositionally variable. The polymeric surface provides abundant basic sites but can lead to nonspecific adsorption and pH-sensitive performance. MPTMS-functionalized silica gels from Johari et al. [39] showed high capacity (102.4 mg g^−1^) through Hg–S bonding, but the well-known oxidative instability of thiols in aqueous mercury systems and the absence of regeneration data limit their utility. In contrast, SG-3PS-Cys achieved a competitive qₘₐₓ = 82.7 mg g^−1^, distinguished by a thiol-free coordination mechanism. The sulfur atom is covalently immobilized within the Si–C–S linkage, while Hg(II) selectively binds via zwitterionic –NH_3_^+^ and –COO^−^ groups, as confirmed by FTIR, XPS, and ^13^C NMR. This design provides enhanced oxidative stability and maintains ≈ 72% regeneration after five cycles. Although its capacity is moderate compared with some sulfur-functionalized systems, its structural robustness, chemical sustainability, and spectroscopically validated mechanism make SG-3PS-Cys a superior reusable candidate for practical Hg(II) remediation in realistic aqueous environments. While this study focused on controlled aqueous systems, the physicochemical resilience of SG-3PS-Cys, particularly its zwitterionic chemistry and thiol-free stability, suggests strong potential for use in complex water matrices such as industrial effluents or contaminated groundwater. Future work will focus on evaluating the performance of SG-3PS-Cys under varying ionic strength and pH, extending its validation to real wastewater conditions beyond the multi-cation experiments performed in this study.

### 2.6. N_2_ Adsorption–Desorption Results

**Figure 7:**
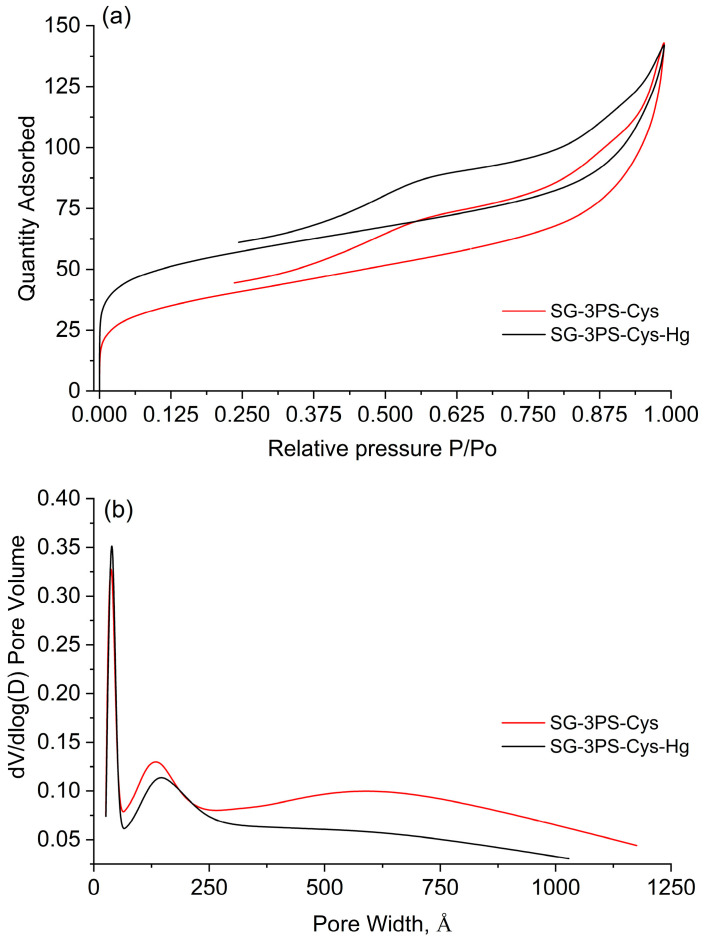
(**a**) Adsorption isotherms of the adsorbent SG-3PS-Cys and SG-3PS-Cys-Hg; (**b**) distributions of pore sizes by BJH method.

Table 4 summarizes the textural parameters of the SG-3PS-Cys and SG-3PS-Cys-Hg adsorbents. SG-3PS-Cys-Hg corresponds to the SG-3PS-Cys material after Hg(II) adsorption. Kinetic, isotherm, and regeneration studies were performed exclusively with SG-3PS-Cys. Afterward, this adsorbent was contacted with a Hg(II) solution, recovered, dried, and characterized to evaluate the structural differences between the material before and after mercury adsorption. The average pore diameter (Dₚ = 9.79 nm) classifies the material as mesoporous, according to the IUPAC definition [41]. The BET surface areas were 134.0 and 188.6 m^2^ g^−1^ for SG-3PS-Cys and SG-3PS-Cys-Hg, respectively, indicating a measurable increase after mercury adsorption. The silica surface contains abundant hydroxyl (–OH) groups that interact strongly with Hg(II) species [42]. These interactions promote partial pore widening and the formation of surface complexes, which increase the effective area available for gas adsorption. The higher BET surface area of SG-3PS-Cys-Hg reflects these structural modifications after Hg(II) uptake. Figure 7a shows the N_2_ adsorption isotherm of SG-3PS-Cys, which corresponds to a type IV(a) profile according to IUPAC classification [41]. The curve exhibits a well-defined H3 hysteresis loop, characteristic of mesoporous materials composed of non-rigid, plate-like aggregates. This hysteresis pattern confirms the presence of slit-shaped pores and interconnected meso- and macropores formed during the sol–gel synthesis. Figure 7b presents the pore-size distribution determined by the BJH method from the desorption branch of the isotherm. The distribution is multimodal with a narrow dominant peak between 2.5 and 5.0 nm, corresponding to uniform mesopores generated by controlled condensation of the silica–cysteine network.

### 2.7. ζ-Potential Results

**Figure 8:**
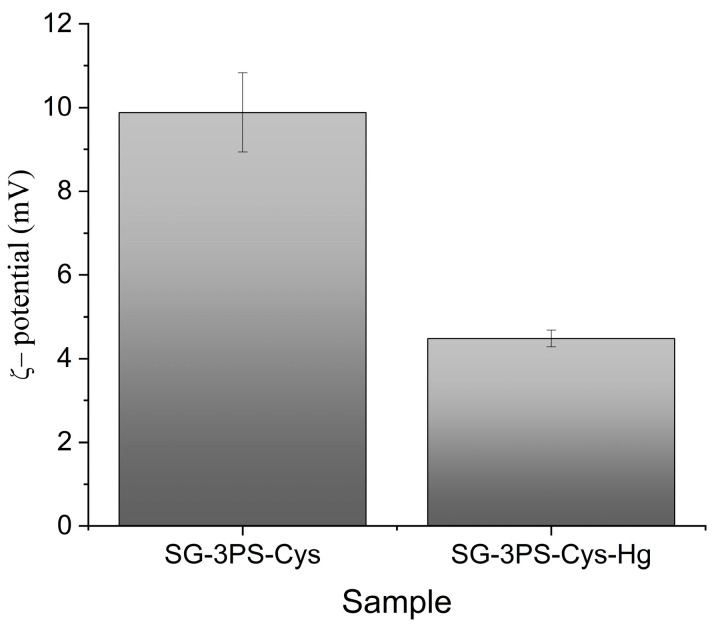
ζ-potential of adsorbent SG-3PS-Cys before and after Hg(II) uptake.

The surface charge behavior of SG-3PS-Cys before and after Hg(II) uptake was evaluated through ζ-potential measurements (Figure 8). The ζ-potential decreased from +9.88 mV to +4.48 mV after Hg(II) uptake, indicating partial neutralization of the positive surface charge due to the approach of anionic Hg–acetate complexes to the surface sites, consistent with electrostatic association rather than direct chemical bonding. Similar ζ-potential decreases have been reported for amine-functionalized silica when inner-sphere complexation occurs with multivalent cations [43].

### 2.8. FTIR Analysis of the SG-3PS-Cys Adsorbent Before and After Hg(II) Uptake

**Figure 9:**
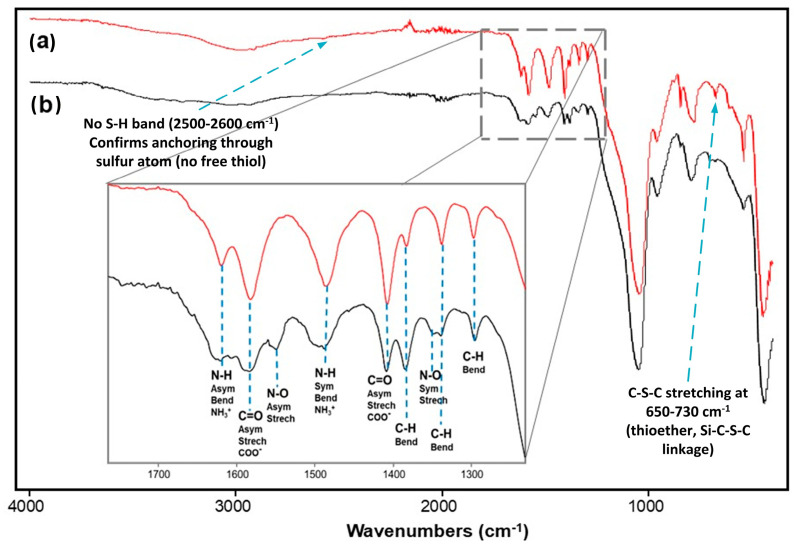
FTIR spectra of the zwitterionic adsorbent before and after mercury uptake: (a) SG-3PS-Cys and (b) SG-3PS-Cys-Hg.

Figure 9 shows the FTIR spectra of SG-3PS-Cys (a) and SG-3PS-Cys-Hg (b). Both spectra display characteristic silica matrix bands at 1050 and 787 cm^−1^, corresponding to Si–O stretching and bending vibrations. The main changes induced by Hg(II) adsorption are observed in the 1700–1200 cm^−1^ region (inset). The SG-3PS-Cys spectrum (Figure 9, (a)) exhibits the zwitterionic fingerprint of cysteine [44]: N–H bending vibrations of the –NH_3_^+^ group at 1621 and 1485 cm^−1^, and COO^−^ asymmetric and symmetric stretching at 1581 and 1408 cm^−1^ [44,45]. Additional bands at 1370, 1335, and 1298 cm^−1^ correspond to the C–H bending of the aliphatic chain. The absence of an S–H stretching band (2550–2600 cm^−1^) confirms covalent anchoring of cysteine through sulfur, forming a thioether linkage (Si–C–S–Cys). A weak band between 650 and 730 cm^−1^, assigned to ν(C–S–C) stretching, further supports thioether formation, consistent with reports of similar C–S–C vibrations near 680–700 cm^−1^ in thioether systems [46]. The absence of an S–H stretching band (2550–2600 cm^−1^) indicates thioether bond formation (Si–C–S–Cys). This interpretation will be further corroborated by XPS analysis (Section 2.11), confirming sulfur covalent anchoring to the silica framework. After mercury uptake (Figure 9, (b)), the spectra exhibit band broadening and intensity reduction in the zwitterionic signals: –NH_3_^+^ (1621, 1448 cm^−1^) and –COO^−^ (1581, 1408 cm^−1^) [47]. New features appear at 1340 cm^−1^, corresponding to the methyl acetate group of mercury acetate, and a shoulder at 1405 cm^−1^, attributed to a bridging carboxylate structure [48,49]. These results propose that Hg(II) binds primarily through the carboxylate groups of cysteine, while ammonium groups contribute to the stabilization of mercury acetate via electrostatic interactions between the zwitterionic centers.

### 2.9. Solid-State NMR Analysis

**Figure 10:**
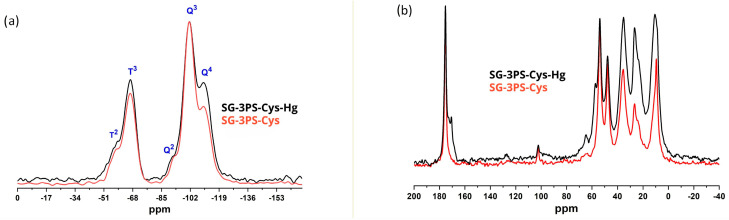
(**a**) ^29^Si CP-MAS NMR spectra of SG-3PS-Cys (red line) and SG-3PS-Cys-Hg (black line). (**b**) ^13^C CP-MAS NMR spectra of SG-3PS-Cys (red line) and SG-3PS-Cys-Hg (black line).

The ^29^Si{^1^H} CPMAS NMR spectra of SG-3PS-Cys and SG-3PS-Cys-Hg show no appreciable differences (Figure 10a). The relative abundance of the silicon environments (T_2_/T_3_/Q_2_/Q_3_ ≈ 10/35/5/50) remains unchanged in both samples, confirming that the silica framework remains structurally stable after Hg(II) interaction. This result indicates that the adsorption process occurs at the organic moieties attached to the surface rather than through disruption of the siloxane network. In contrast, the ^13^C{^1^H} CPMAS NMR spectra (Figure 10b) reveal distinct chemical changes after mercury adsorption. In SG-3PS-Cys, the carboxyl carbon of cysteine resonates at 175.5 ppm, whereas an additional resonance appears at 170.5 ppm in SG-3PS-Cys-Hg, consistent with carboxylate groups involved in metal complexation. The approximate 10:6 ratio of free to metal-associated cysteine indicates that only a fraction of the ligands participates directly in the interaction. This behavior arises from spatial constraints within the mesopores, and electrostatic repulsion created after the first coordination events, limiting access of further Hg(II) species to internal sites. Similar partial participation of amino acid ligands has been reported for mesoporous silicas functionalized with biogenic molecules [50]. The resonance near 55 ppm, corresponding to the α-carbon adjacent to –NH_2_, exhibits partial splitting in SG-3PS-Cys-Hg, suggesting a perturbation of the amine environment due to proximity to coordinated Hg(II). This interpretation aligns with the minor shifts in N–H vibration bands observed in FTIR but does not imply direct coordination through nitrogen. No significant changes were detected between 35 and 45 ppm, where carbons bonded to sulfur appear, confirming that the sulfur atom remains covalently immobilized within the Si–C–S linkage and does not participate in complexation. Overall, the NMR data demonstrate that mercury adsorption produces local electronic rearrangements involving the oxygenated and amino functionalities of cysteine, while preserving the silica network. These findings clarify how the molecular interactions within SG-3PS-Cys govern its selective and stable binding of Hg(II), providing a mechanistic foundation for its reusability and long-term performance in aqueous systems.

### 2.10. XPS Results

**Figure 11:**
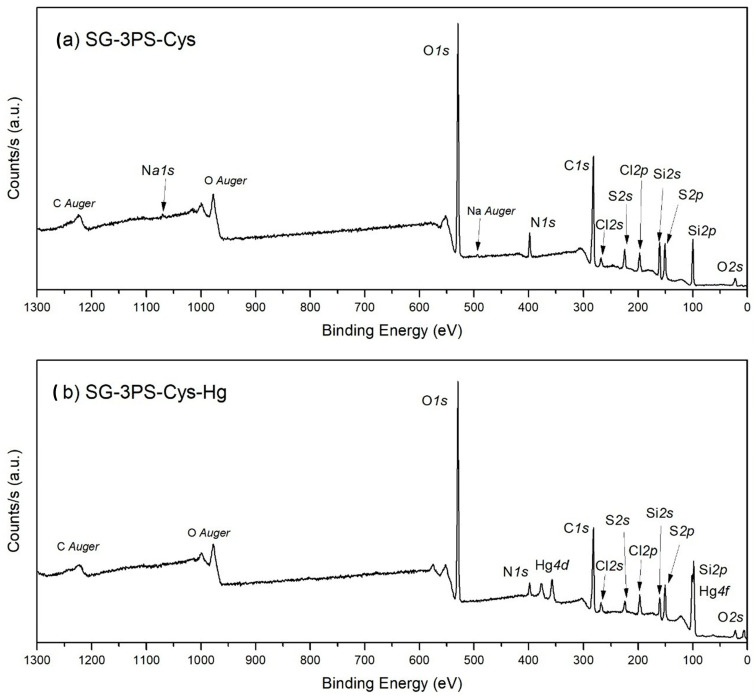
Wide-scan XPS spectra for sample (**a**) SG-3PS-Cys and (**b**) SG-3PS-Cys-Hg.

**Figure 12:**
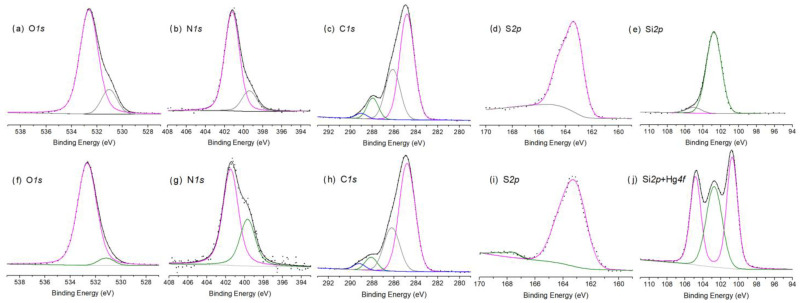
XPS High resolution normalized spectra of O1s, N1s, C1s, S2p and Si2p core levels for sample SG-3PS-Cys (**upper** figures) and SG-3PS-Cys-Hg, respectively, including Hg4f overlapping Si2p in SG-3PS-Cys-Hg sample (**lower** figures).

The wide-scan XPS spectra of SG-3PS-Cys and SG-3PS-Cys-Hg (Figure 11a,b) display the expected O 1s, N 1s, C 1s, S 2p, and Si 2p signals, while the appearance of Hg 4f peaks in the latter confirms successful mercury immobilization. Peak areas were fitted using AAnalyzer v2.25 to determine atomic concentrations (Table 5) [51]; the C 1s region was referenced to C–C = 284.8 eV [52,53]. Minor Na 1s and Cl 2p signals originate from residual salts. After Hg(II) adsorption, new Hg 4f and Hg 4d doublets appear, confirming mercury loading onto the functionalized silica. High-resolution spectra (Figure 12) reveal binding-energy shifts diagnostic of coordination. The O 1s spectrum (Figure 12a,f) shows two contributions from Si–O/C–O and carboxylate oxygen; after Hg(II) uptake, the carboxylate component shifts to lower energy, indicating Hg–O bond formation via charge transfer [54]. The N 1s region (Figure 12b,g) shows a slight decrease in binding energy of the –NH_3_^+^/–NH_2_ species, consistent with electrostatic and hydrogen bond interactions with acetate–ligated mercury rather than direct Hg–N coordination. The C 1s and S 2p spectra (Figure 12c,d,i) confirm that carbon and sulfur remain chemically stable. The C–S signal is unchanged, and the sulfur doublet at ≈163 eV corresponds to thioether, verifying that sulfur is covalently anchored to the silica and does not participate in coordination, consistent with the ^13^C NMR results. The Hg 4f_7_/_2_ (100.7 eV) and Hg 4f_5_/_2_ (104.8 eV) peaks correspond to Hg(II) bound to oxygen donors (Hg–OAc/O–CO), matching reported values for acetate complexes. From an environmental perspective, these findings confirm a chemically robust, non-thiol coordination pathway that maintains the structural stability of the adsorbent and prevents oxidative deactivation. The predominance of Hg–O over Hg–S interactions supports a stable and regenerable adsorption mechanism, highlighting the potential of SG-3PS-Cys for mercury removal in controlled aqueous environments.

#### Mechanism of Hg(II) Uptake and Environmental Relevance

**Figure 13:**
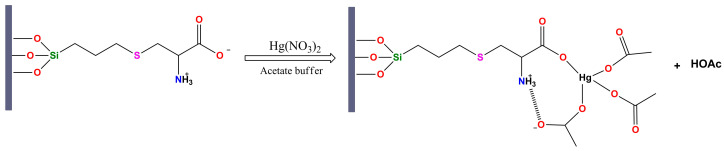
Proposed mechanism of Hg(II) interaction with SG-3PS-Cys. Hg(II) binding in acetate medium (pH ≈ 3): Hg(II) coordinates with acetate and surface carboxylate (–COO^−^), while protonated amines (–NH_3_^+^) electrostatically stabilize the Hg–acetate–carboxylate complex via ion-pairing and hydrogen bonding; no direct Hg–N coordination occurs.

The proposed Hg(II) uptake mechanism was analyzed through MINEQL+ speciation modeling of the Hg(NO_3_)_2_–CH_3_COONa–HNO_3_–H_2_O system [55]. Calculations indicate that at pH ≈ 3, mercury predominantly exists as the anionic complex Hg(Ac)_4_^2−^, stabilized by the acetate buffer. Under these acidic conditions, most surface carboxyl groups remain protonated (–COOH), yet Hg(II)’s strong affinity for oxygen donors induces local deprotonation, forming carboxylate sites (–COO^−^) that establish inner-sphere coordination through Hg–OOC(surface)–OAc bridges [56]. Neighboring –NH_3_^+^ groups electrostatically attract and stabilize Hg(Ac)_4_^2−^ via hydrogen bonding and ion pairing, creating a cooperative zwitterionic configuration (Figure 13). This mechanism acetate-assisted deprotonation coupled with cation–anion stabilization is more consistent with acidic media than direct Hg–N bonding. The model coherently explains the Hg–O coordination observed in FTIR and XPS and the reproducible Hg(II) uptake despite the absence of thiol groups. Although acetate is not dominant in natural waters, this controlled system clarifies the intrinsic coordination chemistry of the zwitterionic surface. The insight is transferable to environments where Hg(II) occurs as complexed species (e.g., Hg–chloride or Hg–organic ligands), providing a predictive basis for sorbent behavior. This mechanism is particularly relevant for aqueous systems containing acetate or other organic acids that influence mercury speciation. The demonstrated ability of SG-3PS-Cys to capture Hg(II) efficiently in such controlled conditions without relying on reactive thiol groups highlights its oxidation resistance, recyclability, and reduced tendency toward irreversible binding. The absence of permanent Hg–S bonds facilitates straightforward regeneration and supports the potential of this zwitterionic pathway as a sustainable alternative for future water treatment studies.

### 2.11. Surface Energy Distribution, Porous Structure, and Zeta Potential: Synergistic Influence on Mercury Uptake

The surface energy distribution (SED) connects the microscopic heterogeneity of an adsorbent to its macroscopic performance. Each functional group on the SG-3PS-Cys surface possesses a characteristic adsorption energy defining its interaction with Hg(II). The DFT-derived SED links this local heterogeneity to overall adsorption behavior: uniform energy profiles yield faster, predictable kinetics, while broader distributions produce stepwise adsorption and gradual site filling. Thus, SED bridges molecular-scale chemistry with properties such as capacity, regeneration, and long-term stability. DFT analysis of N_2_ adsorption–desorption data quantifies energetic domains associated with specific functional groups, complementing textural characterization by describing the energetic landscape that governs selectivity and stability in water treatment systems.

**Figure 14:**
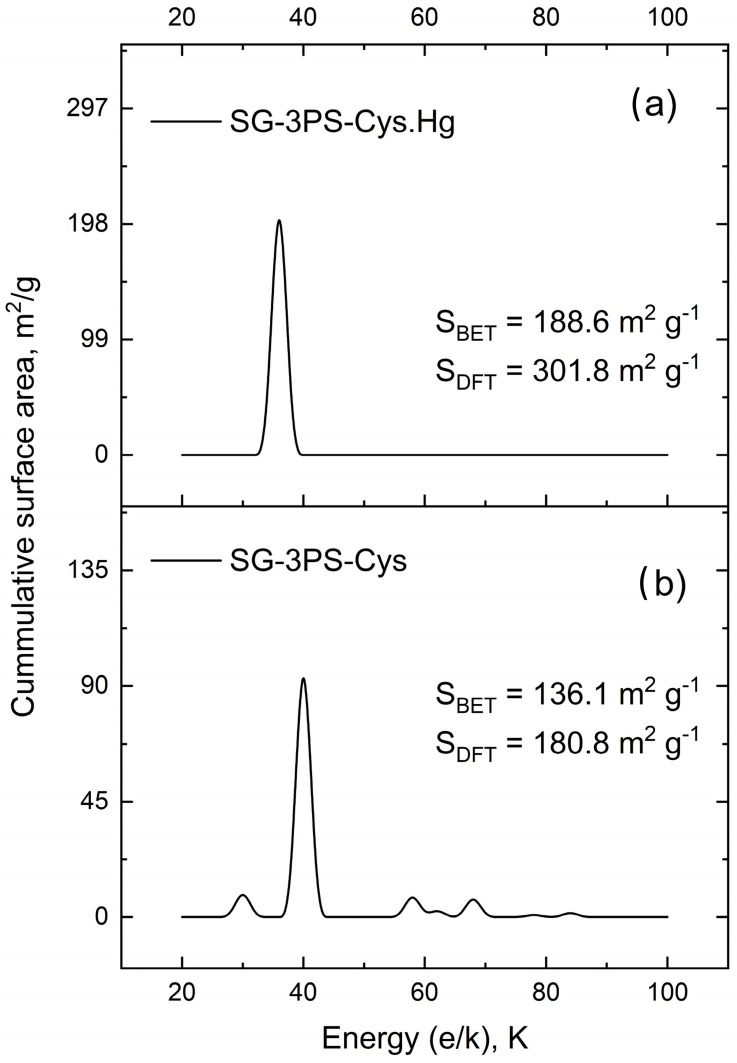
Comparison of surface energy distributions and specific surface areas derived from BET and DFT analyses for (**a**) SG-3PS-Cys-Hg and (**b**) SG-3PS-Cys adsorbents.

The dominant energy peak at ε/k ≈ 38 K corresponds to low-energy adsorption sites characteristic of mesoporous materials, while minor shoulders indicate slightly higher-energy domains (Figure 14b). Given the purely mesoporous nature of the material, the energy distribution function obtained from N_2_ adsorption reflects changes in mesoporous surface energetic heterogeneity rather than ultramicropore-related effects. These features imply a largely homogeneous surface with limited heterogeneity. The correlation between these energetic domains and specific functional groups is supported by the previously discussed FTIR, XPS, and NMR results, which collectively confirm the presence of stable, zwitterionic surface functionalities contributing to Hg(II) capture. Energies below 100 K correspond to weak van der Waals physisorption on mesoporous surfaces, consistent with a uniform arrangement of zwitterionic moieties. Broader or higher-energy peaks (>1000 K) reported elsewhere reflect heterogeneity and chemisorption, which are absent here. The material retains a mesoporous structure (Dₚ ≈ 9.8 nm), type IV(a) isotherm with H3 hysteresis [41], although functionalization can produce confined high-energy domains without structural microporosity. Minor peaks at 22–30 K for SG-3PS-Cys suggest secondary low-energy sites, attributed to unfunctionalized silanols, propyl chains, or misoriented zwitterionic groups typical of partially functionalized silicas [41,57]. Weak high-energy shoulders (>40 K) reflect localized domains arising from –NH_3_^+^/–COO^−^ cooperation or steric confinement, enhancing Hg(II) affinity [58]. Similar multimodal SEDs occur in hybrid silicas with heterogeneous pore-wall chemistry or partial collapse [59].

After Hg(II) adsorption, the DFT surface area increased from 180.8 to 301.8 m^2^ g^−1^ and the BET area from 136.1 to 188.6 m^2^ g^−1^ (Figure 14a). Here, SG-3PS-Cys-Hg corresponds to the cysteine-functionalized mesoporous silica after Hg(II) adsorption under equilibrium conditions, as described in Section 2.6. Although uncommon, this may result from partial pore unblocking and wettability changes caused by the acetate medium and drying, consistent with SBA-15 solvent-exposure studies and metrological specific surface area guidelines [59,60]. Simultaneously, the ζ-potential decreased from +9.88 to +4.48 mV, indicating surface charge reduction due to electrostatic association between Hg(II)–acetate complexes and –NH_3_^+^ groups, which promotes denser packing and improved access to high-energy sites. Comparable charge–porosity interdependence has been reported for hybrid materials where ζ-potential modulates aggregation and reactivity [61].

The SED findings clarify how adsorption energy and surface charge govern pollutant capture and reusability. The combined increase in accessible surface area, energetic uniformity, and adaptive ζ-potential explains the stable and regenerative Hg(II) uptake of SG-3PS-Cys. Collectively, these structural and energetic features demonstrate the material’s durability, efficiency, and sustainability for future mercury removal applications in aqueous systems.

## 3. Conclusions

A durable and regenerable mercury adsorbent (SG-3PS-Cys) was synthesized by covalently anchoring L-cysteine onto a mesoporous silica matrix through a propyl-silane linker. Unlike conventional thiol-based materials, SG-3PS-Cys immobilizes sulfur within the framework, enabling selective Hg(II) binding through zwitterionic amino and carboxylate groups. FTIR, solid-state ^13^C NMR, and XPS confirmed a thiol-free coordination pathway that provides enhanced chemical stability and oxidative resistance critical for long-term water treatment applications. The adsorbent exhibited rapid Hg(II) uptake following pseudo-second-order kinetics and retained over 70% removal efficiency after multiple regeneration cycles using simple alkaline washing. ζ-potential analysis revealed partial neutralization of surface –NH_3_^+^ groups upon Hg(II) coordination, corroborating the electrostatic contribution to zwitterionic complexation and the maintenance of colloidal stability during reuse. DFT-based surface energy distribution analysis showed homogeneous, low-energy adsorption domains associated with –NH_3_^+^/–COO^−^ groups, consistent with selective and reversible Hg(II) capture. The synergy between mesoporosity, surface energetics, and electrostatic charge adaptation explains the material’s high reusability and stability.

Although tested under controlled acetate-buffered conditions, the robustness and zwitterionic nature of SG-3PS-Cys suggest strong potential for future validation in complex and multi-ion water matrices. Overall, this study establishes a chemically resilient, thiol-free adsorbent that integrates mesoporosity, surface charge dynamics, and zwitterionic coordination, offering a sustainable design framework for next-generation materials in mercury remediation.

## 4. Materials and Methods

### 4.1. Materials

Tetraethyl orthosilicate (TEOS, 99%) was used as the main silica source, L-cysteine (Cys, 98%) as the active ligand, and (3-chloropropyl) triethoxysilane (3CPTES, 97%) as the coupling agent. Mercury (II) nitrate monohydrate (≥98%) was used for preparing Hg solutions, and triethylamine (TEA, 99.5%) served as the gelling agent. All reagents were purchased from Sigma-Aldrich (Toluca, Mexico) and used without further purification. Nitric acid, absolute ethanol (EtOH), and NaCl were obtained from Golden Bell,, Mexico.

### 4.2. Synthesis of the Functional Precursor (3PTES-Cys)

**Figure 15:**
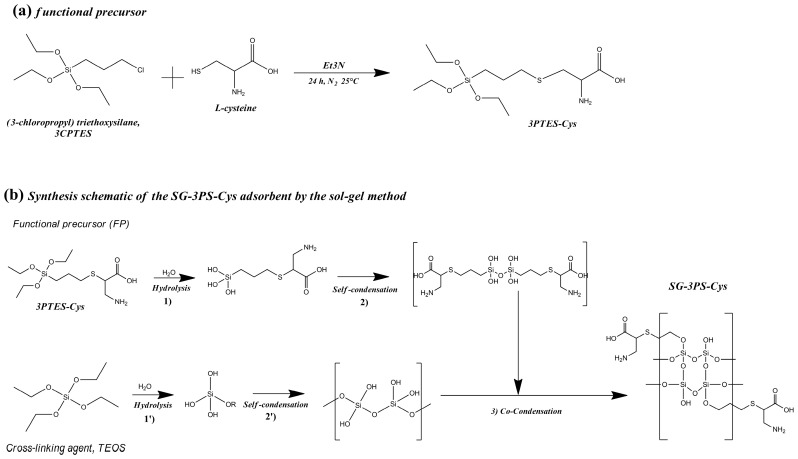
(**a**) Synthesis of the functional precursor (FP). (**b**) Schematic of the synthesis of the SG-3PS-Cys adsorbent by the sol–gel method [62].

The functional precursor was synthesized following the sol–gel-based method of Moran-Salazar et al. [62], with minor modifications. Briefly, L-cysteine was dissolved in a water/ethanol mixture (molar ratio Cys:H_2_O:EtOH = 1:27:8) in a three-neck flask. Triethylamine (TEA) and 3CPTES were then added dropwise in equimolar amounts under a nitrogen atmosphere to prevent premature hydrolysis. The reaction was stirred for 24 h at room temperature, during which the chlorine atom of 3CPTES reacted with the thiol group of cysteine to form a thioether bond, yielding the precursor 3PTES-Cys (3-propyltriethoxysilane-cysteine). This compound retains the amino (–NH_2_) and carboxylate (–COOH) groups of cysteine available for metal coordination, while the thiol (–SH) is covalently linked to the propyl silane moiety (Figure 15a).

### 4.3. Synthesis of the Cysteine-Functionalized Silica Adsorbent (SG-3PS-Cys)

The functionalized silica, SG-3PS-Cys, was obtained by co-condensation of TEOS and 3PTES-Cys via a modified sol–gel route. Hydrolysis and condensation of 3PTES-Cys (steps 1 and 2, Figure 15b) were performed under continuous stirring for 24 h at 298 K. Separately, a silica sol was prepared by mixing TEOS, ethanol, NaCl, and water in a molar ratio of 4:16:0.04:16, adjusting the pH to 11 with TEA to initiate hydrolysis (steps 1′ and 2′, Figure 15b). NaCl was employed to adjust the ionic strength of the reaction medium during TEOS hydrolysis. The presence of Na^+^ and Cl^−^ ions modifies electrostatic interactions among silanol species, affecting hydrolysis condensation kinetics and promoting controlled network formation, without acting as a catalytic [63]. Both soils were then combined at a TEOS:3PTES-Cys molar ratio of 4:1 and stirred for 24 h at 298 K to promote co-condensation (step 3, Figure 15b), forming a gel under basic conditions. The solid was aged 48 h at room temperature, dried at 50 °C for 48 h, and sequentially washed with water and acetone (3 mL g^−1^, three cycles) using vortex agitation and centrifugation. After a final rinse with deionized water, the material was dried again (50 °C, 48 h), ground, and sieved to obtain 125–180 µm particles, minimizing external mass-transfer resistances [64]. The final product, denoted SG-3PS-Cys, corresponds to a silica gel bearing the covalently linked 3-propylsilane-cysteine (Figure 15b). The final product, denoted SG-3PS-Cys, corresponds to a silica gel bearing the covalently linked 3-propylsilane-cysteine (Figure 15b).

### 4.4. Hg(II) Adsorption Kinetics

The adsorption kinetics of Hg(II) on SG-3PS-Cys were evaluated at three initial concentrations (C_0_ = 93, 318, and 593 mg L^−1^), representing low, intermediate, and high driving-force conditions. Independent triplicate suspensions (*n* = 3) of 20 mL solutions (pH 3; 298 K; 0.1 M acetate buffer; 0.1 M ionic strength) were contacted with 0.2 g of SG-3PS-Cys (10 g L^−1^) for 1–480 min. This dosage was selected to ensure measurable concentration changes and accurate determination of equilibrium times and kinetic constants. After agitation in a thermostated bath, samples were filtered and analyzed for total Hg by ICP- MS (Agilent 7500a). Error bars represent ±1 SD, and kinetic parameters are expressed as mean ± 95% Confidence Interval obtained by nonlinear regression (OriginPro 2024 V 10.1, Levenberg–Marquardt algorithm). Model evaluation used both the coefficient of determination (R^2^) and the Akaike Information Criterion (AIC) [65] AIC = *n* ln (RSS/n) + 2*k*, where *n* = number of data points, *k* = number of adjustable parameters, and *RSS* = residual sum of squares. Relative model likelihoods were ranked by ΔAIC (ΔAIC < 2 = equivalent; 4–7 = less supported; >10 = unsupported). The amount of Hg(II) adsorbed was determined as follows:

(1) $$q=\frac{V\left(C_{i}-C_{f}\right)}{m}$$

where:

*q* = amount of Hg (II) adsorbed (mg g^−1^), *V* = solution volume (L), *C_i_* = initial Hg (II) concentration (mg L^−1^), *C_f_* = final Hg (II) concentration (mg L^−1^), *m* = mass of adsorbent (g).

### 4.5. Equilibrium Adsorption Isotherms

Batch equilibrium experiments were conducted to determine the maximum Hg(II) uptake at pH 3–5, representative of wastewater effluents [37]. Initial concentrations ranged from 50 to 1500 mg L^−1^, prepared by diluting a 2 g L^−1^ stock solution at constant pH. A 0.1 M sodium-acetate buffer-maintained pH stability and prevented Hg(OH)_2_ precipitation while preserving the adsorbent’s protonation state. Approximately 0.2 g of SG-3PS-Cys was contacted with 20 mL of Hg(II) solution and agitated for 240 min at 298 K. After filtration, mercury concentrations were measured by ICP-MS, and equilibrium uptake was calculated with Equation (1). Reported values correspond to the mean of three replicates. Nonlinear regressions (OriginPro 2024 V 10.1, Levenberg–Marquardt) were used to fit the isotherm models.

### 4.6. Selectivity Toward Multi-Cation Solutions

The adsorbent’s affinity for multiple cations (Cr, Fe, Cd, Pb, Cu, Hg) was tested under sub-monolayer coverage to assess intrinsic binding affinities. The SG-3PS-Cys surface was preloaded to ~15% of its total capacity (0.343 mmol N g^−1^ by elemental analysis; θ ≈ 0.15) to ensure near-Henry-law conditions (Σb_i_C_i_ < 1; C_i_ is the aqueous concentration of species *i*, (e.g., Hg^2+^). *b*_*i*_ is the adsorption affinity constant of species *i*, which reflects the strength of the interaction between the adsorbent surface and that species, ensuring that metal uptake ratios reflect intrinsic binding affinities rather than saturation effects to study the reactivity of cysteine functional groups toward the cationic species. A 50 mL aliquot of a mixed solution containing 0.0489 mmol of each cation was contacted with 0.2 g of SG-3PS-Cys adsorbent (pH 3, 298 K) for 24 h under stirring. After filtration, metal concentrations were determined using an Analytik Jena ContrAA 300 AAS (for Cr, Fe, Cd, Pb, Cu) and ICP-MS (for Hg). Calibration employed certified standards (Sigma-Aldrich, Toluca, Mexico). The removal percentage was obtained as follows:

(2) $$removal\;\%=\frac{Co-C_{e}}{Co}*100$$

where *C_o_* and *C_e_* are the initial and final cation solution concentrations, respectively (mg L^−1^).

### 4.7. Stability and Regeneration

The reusability of SG-3PS-Cys was tested over five adsorption–desorption cycles. In each cycle, 0.5 g of adsorbent was contacted with 25 mL of 1000 mg L^−1^ Hg(II) solution (pH 3, 298 K, 0.1 M sodium-acetate buffer) for 24 h under stirring. Post-adsorption, the solid was filtered, washed with 0.1 M NaOH and deionized water until neutral pH, dried (50 °C, 24 h), and reused. Mercury content was determined by ICP-MS, and the amounts adsorbed and removed were calculated via Equations (1) and (2), respectively. This cyclic test evaluated the structural stability and regeneration performance of the adsorbent under acidic conditions, confirming its chemical robustness and potential for repeated use in water treatment applications.

### 4.8. Physicochemical Characterization of SG-3PS-Cys

Textural properties (specific surface area, pore volume, and pore size distribution) were determined by N_2_ adsorption–desorption at 77 K using an ASAP 2020 KMP sorptometer (Micromeritics, Norcross, GA, USA). Prior to analysis, 0.2 g of sample was degassed under vacuum (≈10 µm Hg) at 373 K for 12 h to remove adsorbed moisture and CO_2_. The BET equation was applied within 0.05 < *p*/*p*_0_ < 0.30 to calculate surface area, while total pore volume was obtained at *p*/*p*_0_ = 0.995. Pore size and distribution were derived from the desorption branch using the Barrett–Joyner–Halenda (BJH) method based on the desorption branch of the isotherm. The electrophoretic mobility of SG-3PS-Cys suspensions was measured with a Malvern Zetasizer (Worcestershire, UK) and converted to ζ-potential using the Smoluchowski equation.

### 4.9. FTIR Analysis

Fourier-transform infrared spectra were recorded to identify the amino acid functional groups before and after Hg(II) adsorption, using a Thermo Scientific Nicolet iS5 spectrometer equipped with an ATR iD5 module (Waltham, MA, USA). Prior to analysis, samples were dried at 50 °C for 24 h. Spectra were collected over 500–4000 cm^−1^ with 4 cm^−1^ resolution and 16 scans per sample.

### 4.10. Solid-State NMR Spectroscopy

Samples were dehydrated for 48 h at 333 K before analysis. Solid-state NMR was performed at room temperature on an Agilent (Santa Clara, CA, USA) Infinityplus spectrometer (7 T) equipped with a variable-temperature Chemagnetics-Varian 6 mm CPMAS probe. The ^13^C{^1^H} CPMAS NMR spectra were acquired with a 3 ms cross-polarization time, 90° pulse (5.0 µs), 5 s relaxation delay, and 7 kHz MAS rate. The ^29^Si{^1^H} CPMAS NMR spectra were recorded under similar conditions with a 5 ms contact time. Although this technique cannot precisely determine Q^3^/Q^4^ ratios (due to short relaxation), longer delays (≈180 s, 15° pulse) are typically required [66,67]. However, ^29^Si{^1^H} CPMAS enables reliable identification of Q^2^, Q^3^, T^1^ and T^2^ species owing to its high signal-to-noise ratio [68,69]. Spectral deconvolution and numerical integration were carried out using WSolids1 software [70].

### 4.11. X-Ray Photoelectron Spectroscopy (XPS)

Surface chemical states and Hg(II) coordination on SG-3PS-Cys were analyzed by XPS using a Phoibos 150 analyzer (SPECS, Berlin, Germany) with a monochromatic Al Kα source (hν = 1486.7 eV, 250 W). Survey spectra were collected at 100 eV pass energy with 0.5 eV steps, and high-resolution scans at 30 eV pass energy with 0.1 eV steps. The main-chamber pressure was maintained at 2.3 × 10^−9^ Torr during all analyses.

### 4.12. Surface Energy Distribution Analysis

The surface energy distribution (SED) provides a quantitative description of how adsorption energy varies across a porous surface, reflecting the strength of interactions between adsorbate molecules (e.g., N_2_) and heterogeneous surface sites [17]. Unlike BET analysis, which assumes uniform adsorption energy, SED reveals the energetic heterogeneity of the adsorbent, information essential to understanding its chemical reactivity and affinity toward pollutants such as heavy metals. In this study, SED profiles were derived from N_2_ adsorption isotherms using MicroActive v6.0 software (Micromeritics Inc., USA), based on density functional theory (DFT). The model assumes slit-pore geometry, appropriate for mesoporous silica, and correlates each portion of the desorption isotherm to a corresponding adsorption–energy interval, generating a detailed energetic map of the surface. This approach links physicochemical characterization with environmental functionality, enabling the identification of potential high-energy adsorption domains associated with the functional groups introduced by L-cysteine. Therefore, SED analysis not only quantifies surface heterogeneity but also offers mechanistic insight that can guide predictions of long-term performance in future wastewater treatment applications.

## Figures and Tables

**Table 1:** Fitting results of kinetic models for Hg(II) adsorption on SG-3PS-Cys.

Model/Parameters	Initial Solution Concentration, mg L^−1^			Equation
	93	318	593	
q_max exp_ (mg g^−1^)	4.05	19.97	35.11	
Pseudo-first order				
q_max calc_ (mg g^−1^)	4.13 ± 0.18	17.86 ± 0.52	33.28 ± 0.85	$q=q_{e}\left(1-e^{-kt}\right)$
*k*_1_ (min^−1^)	0.007 ± 0.001	0.039 ± 0.002	0.112 ± 0.004	
R^2^	0.933	0.882	0.920	
AIC/ΔAIC	−58.8/10.9	−43.9/10.4	−53.3/3.1	
Pseudo-second order				
q_max calc_ (mg g^−1^)	5.64 ± 0.23	19.80 ±0.47	35.49 ± 0.92	$q=\frac{k_{2}q_{e}^{2}t}{1+k_{2}q_{e}^{2}t}$
*k*_2_ (g mg min^−1^)	0.001 ± 0.0001	0.002 ± 0.0002	0.004 ± 0.0003	
R^2^	0.927	0.919	0.923	
AIC/ΔAIC	−58.6/11.1	−54.3/0.0	−53.9/2.5	
Intraparticle diffusion				
A (mg g^−1^ min^−1/2^)	0.226 ± 0.012	0.874 ± 0.029	1.237 ± 0.041	$q=A\;t^{1/2}+B$
B (mg g^−1^ min^−1^)	−0.502 ± 0.030	3.413 ± 0.042	14.19 ± 0.06	
R^2^	0.903	0.763	0.569	
AIC/ΔAIC	−49.6/20.1	−25.4/28.9	−8.7/47.7	
Avrami				
q_max calc_ (mg g^−1^)	3.47 ± 0.20	18.3 ± 0.45	32.8 ± 0.71	$q=q_{e}\left(1-e^{(-k_{av}t^{n_{av}}}\right)$
K_a_ (min^−1^)	0.037 ± 0.002	0.019 ± 0.002	0.172 ± 0.010	
N_a_	7.148 ± 0.218	0.642 ± 0.050	1.573 ± 0.081	
R^2^	0.971	0.895	0.940	
AIC/ΔAIC	−69.7/0.0	−47.4/6.9	−56.4/0.0	

Notes: Uncertainties are ±95% Confidence Interval from nonlinear regressions using the Levenberg–Marquardt algorithm; AIC = *n* ln(RSS/n) + 2k; ΔAIC = AIC_i_ − AIC_min_; lowest AIC = best fit.

**Table 2:** Results of data adjustments to the models of Hg(II) ions adsorption isotherms on the adsorbent SG-3PS-Cys.

**Models**	**Initial Solution pH**			**Equation**
	**3**	**4**	**5**	
**q_max exp_ (mg g^−1^)**	82.67	33.61	31.98	
**Lagmuir**				
**q_max_ (mg g^−1^)**	82.01	32	33	$q=\frac{Kq_{max}C}{1+KC}$
**K (L mg^−1^)**	0.009	0.002	0.002	
**R^2^**	0.677	0.628	0.726	
**Freundlich**				
**K_f_ (mg^1−1/n^ L^1/n^ g^−1^)**	1.408	0.420	0.524	$q=K_{f}{C_{e}}^{1/n}$
**n**	1.501	1.700	1.800	
**R^2^**	0.786	0.716	0.771	
**Toth**				
**q_max_ (mg g^−1^)**	82.01	32	33	$q=\frac{q_{max}C}{{\left(a_{T}+C\right)}^{1/t}}$
**aT (L/mg)**	0.004	0.0008	0.0008	
**t**	8.136	−4597	−6 × 10^6^	
**R^2^**	0.911	−0.034	−0.437	
**S-shape**				
**No (mg g^−1^)**	74.47	32.43	33.56	$q=\frac{N_{0}}{\left(1+k_{2}e^{\left(-k_{1}C\right)}\right)}$
**k_1_**	0.038	0.013	0.006	
**k_2_**	303.8	13,314	56.69	
**R^2^**	0.989	0.957	0.970	
**Langmuir two sites**				
**q_max1_ (mg g^−1^)**	82	0.0002	2041	$q=\frac{q_{max1}b_{1}C}{\left(1+b_{1}C\right)}+\frac{q_{max}b_{2}C}{\left(1+b_{2}C\right)}$
**q_max2_ (mg g^−1^)**	5	3.555	−1.869	
**b_1_**	0.007	−1.2 × 10^−10^	1.5 × 10^−5^	
**b_2_**	0.050	−9 × 10^44^	4.7 × 10^46^	
**R^2^**	0.700	−0.808	0.933	
**Freundlich-Langmuir**				
**q_max1_ (mg g^−1^)**	80.73	32.53	36.82	$q=\frac{q_{max}{\left(K_{FL}C\right)}^{1/n}}{\left(1+{\left(K_{FL}C\right)}^{1/n}\right)}$
**K_FL_**	0.007	0.002	0.002	
**n**	0.225	0.108	0.285	
**R^2^**	0.995	0.952	0.956	

**Table 3:** Comparative performance and mechanistic characteristics of representative Hg(II) adsorbents reported in the literature.

Adsorbent	Functional Group	qₘₐₓ (mg/g)	Binding Mechanism	Regeneration	Reference
MPTMS MCM 41–NH_2_	Aminopropyl (–NH_2_) grafted on MCM 41	125	Hg–N coordination	Yes (10 cycles, water regeneration)	[35]
Cys C@Fe_3_O_4_ (carbon-coated magnetite)	–SH, –NH_2_, –COOH	94.3	Hg–S/N coordination	Yes (3 cycles)	[36]
Hydride silica composites	–Si–H (hydride) groups	~101	Hg–Si/H interactions	Not evaluated	[40]
PEI modified tobermorite	Polyethyleneimine (–NH_2_-rich)	82.6	Hg–N chelation	Yes	[38]
MPTMS-functionalized silica	–SH (MPTMS grafted)	102.4	Hg–S binding	Not specified	[39]
SG-3PS-Cys silica (no free –SH)	–NH_3_^+^, –COO^−^ (no –SH)	82.7	Hg–N/Hg–O (zwitterionic, thiol-free)	Yes (~72% retained after 5 cycles)	This work

**Table 4:** Results of N_2_ adsorption–desorption by the BET method.

Sample	S_BET_ (m^2^ g^−1^)	C_BET_	V_p_ (cm^3^ g^−1^)	D_p_ (nm)
SG-3PS-Cys	134.0	433.7	0.181	9.8
SG-3PS-Cys-Hg	188.6	1344	0.152	7.1

**Table 5:** Atomic concentrations are calculated as organic compounds.

Sample	O1*s*, C1*s*, Si2*p*	N1*s*	S2*p*	Hg4*f*
SG-3PS-Cys	89.0%	3.9%	7.1%	--
SG-3PS-Cys-Hg	88.8%	3.0%	6.1%	2.1%

## Data Availability

Data available upon request.

## References

[1] Selin N.E. (2009). Global Biogeochemical Cycling of Mercury: A Review. Annu. Rev. Environ. Resour..

[2] Kismelyeva S., Khalikhan R., Torezhan A., Kumisbek A., Akimzhanova Z., Karaca F., Guney M. (2021). Potential Human Exposure to Mercury (Hg) in a Chlor-Alkali Plant Impacted Zone: Risk Characterization Using Updated Site Assessment Data. Sustainability.

[3] Horowitz H.M., Jacob D.J., Amos H.M., Streets D.G., Sunderland E.M. (2014). Historical Mercury Releases from Commercial Products: Global Environmental Implications. Environ. Sci. Technol..

[4] van Velzen D., Langenkamp H., Herb G. (2002). Review: Mercury in waste incineration. Waste Manag. Res. J. A Sustain. Circ. Econ..

[5] WHO (2005). Mercury in Drinking-Water Background Document for Development of WHO Guidelines for Drinking-Water Quality. https://www.who.int/docs/default-source/wash-documents/wash-chemicals/mercury-background-document.pdf.

[6] UNEP (2019). Global Mercury Assessment. https://www.unep.org/resources/publication/global-mercury-assessment-2018.

[7] Azimi A., Azari A., Rezakazemi M., Ansarpour M. (2017). Removal of Heavy Metals from Industrial Wastewaters: A Review. ChemBioEng Rev..

[8] Jyothi N.R., Farook N.A.M. (2020). Mercury Toxicity in Public Health. Heavy Metal Toxicity in Public Health.

[9] Cheng X.Z., Hu C.J., Cheng K., Wei B.M., Hu S.C. (2010). Removal of mercury from wastewater by adsorption using thiol-functionalized eggshell membrane. Adv. Mater. Res..

[10] Himanshu Agarwal H.A., Divyanshi Sharma D.S., Sindhu S.K., Sonika Tyagi S.T., Saiqa Ikram S.I. (2010). Removal of mercury from wastewater use of green adsorbents-a review. Electron. J. Environ. Agric. Food Chem..

[11] Agency for Toxic Substances and Disease Registry (2022). Toxicological Profile for Mercury Draft for Public Comment. https://wwwn.cdc.gov/TSP/ToxProfiles/ToxProfiles.aspx?id=115&tid=24.

[12] Liang R., Zou H. (2020). Removal of aqueous Hg(II) by thiol-functionalized nonporous silica microspheres prepared by one-step sol–gel method. RSC Adv..

[13] Liu X., Wu M., Li C., Yu P., Feng S., Li Y., Zhang Q. (2022). Interaction Structure and Affinity of Zwitterionic Amino Acids with Important Metal Cations (Cd2+, Cu2+, Fe3+, Hg2+, Mn2+, Ni2+ and Zn2+) in Aqueous Solution: A Theoretical Study. Molecules.

[14] United States Environmental Protection Agency (2001). Water Quality Criterion for the Protection of Human Health.

[15] World Health Organization (2004). Guidelines for Drinking-Water Quality.

[16] Yang R., Liu G., Li M. (2010). Analysis of the effect of drying conditions on the structural and surface heterogeneity of silica aerogels and xerogel by using cryogenic nitrogen adsorption characterization. Microporous Mesoporous Mater..

[17] Micromeritics Intrument Corp (2018). Application Note: Surface Energy Distribution by DFT Analysis of Nitrogen Adsorption Data. https://www.micromeritics.com.

[18] Diario Oficial de la federación (2021). NOM- 001-SEMARNAT 2021. Que establece los límites permisibles de contaminantes en las descargas de aguas residuals en cuerpos receptores propiedad de la nación. Mexico. https://www.cofemersimir.gob.mx/portales/resumen/49271.

[19] Stong T., Osuna C.A., Shear H., Sanchez J.d.A., Ramírez G., Torres J.d.J.D. (2013). Mercury concentrations in common carp (Cyprinus carpio) in Lake Chapala, Mexico: A lakewide survey. J. Environ. Sci. Health A Tox. Hazard. Subst. Environ. Eng..

[20] Torres Z., Mora M.A., Taylor R.J., Alvarez-Bernal D., Buelna H.R., Hyodo A. (2014). Accumulation and hazard assessment of mercury to waterbirds at Lake Chapala, Mexico. Environ. Sci. Technol..

[21] Silva A.d.S.d., de Moraes D.P., dos Santos J.H.Z. (2019). Sol-gel hybrid silicas as an useful tool to mercury removal. J. Environ. Chem. Eng..

[22] Wei S., Guo C., Wang L., Xu J., Dong H. (2021). Bacterial synthesis of PbS nanocrystallites in one-step with l-cysteine serving as both sulfur source and capping ligand. Sci. Rep..

[23] Adnan S., Kalwar N.H., Abbas M.W., Soomro R.A., Saand M.A., Awan F.R., Avci A., Pehlivan E., Bajwa S. (2019). Enzyme-free colorimetric sensing of glucose using l-cysteine functionalized silver nanoparticles. SN Appl. Sci..

[24] Tomar D., Kaur H., Kaur H., Rana B. (2019). ATR-FTIR Spectroscopy and Its Relevance to Probe the Molecular-Level Interactions Between Amino Acids and Metal-Oxide Nanoparticles at Solid/Aqueous Interface.

[25] Rodríguez-De-La-Peña S., Gómez-Salazar S., Gutiérrez-Ortega J.A., Badillo-Camacho J., Peregrina-Lucano A.A., Shenderovich I.G., Manríquez-González R. (2022). Novel Silica Hybrid Adsorbent Functionalized with L-Glutathione Used for the Uptake of As(V) from Aqueous Media. Ind. Eng. Chem. Res..

[26] Mohan D., Gupta V., Srivastava S., Chander S. (2001). Kinetics of mercury adsorption from wastewater using activated carbon derived from fertilizer waste. Colloids Surfaces A Physicochem. Eng. Asp..

[27] Hu Q., Ma S., He Z., Liu H., Pei X. (2024). A revisit on intraparticle diffusion models with analytical solutions: Underlying assumption, application scope and solving method. J. Water Process. Eng..

[28] Plazinski W., Rudzinski W., Plazinska A. (2009). Theoretical models of sorption kinetics including a surface reaction mechanism: A review. Adv. Colloid Interface Sci..

[29] Liu Z., Sun Y., Xu X., Qu J., Qu B. (2020). Adsorption of Hg(II) in an Aqueous Solution by Activated Carbon Prepared from Rice Husk Using KOH Activation. ACS Omega.

[30] Tchobanoglous G., Burton F.L., Stensel H.D. (2003). Wastewater Engineering Treatment and Reuse.

[31] Smith R.M., Martell A.E., Motekaitis R.J. (2004). Critically Selected Stability Constants of Metal Complexes. https://www.nist.gov/system/files/documents/srd/46_8.pdf.

[32] Giles C.H., MacEwan T.H., Nakhwa S.N., Smith D. (1960). Studies in adsorption. Part XI. A system of classification of solution adsorption isotherms, and its use in diagnosis of adsorption mechanisms and in measurement of specific surface areas of solids. J. Chem. Soc..

[33] Algieri V., Tursi A., Costanzo P., Maiuolo L., De Nino A., Nucera A., Castriota M., De Luca O., Papagno M., Caruso T. (2024). Thiol-functionalized cellulose for mercury polluted water remediation: Synthesis and study of the adsorption properties. Chemosphere.

[34] Frank P., Sarangi R., Hedman B., Hodgson K.O. (2019). Synchrotron X-radiolysis of L-cysteine at the sulfur K-edge: Sulfurous products, experimental surprises, and dioxygen as an oxidoreductant. J. Chem. Phys..

[35] Hamid S., Syed W., Mohammad G. (2009). Synthesis and Characterization of Amino-functionalized Meso- porous Silicate MCM-41 for Removal of Toxic Metal Ions. Chin. J. Chem..

[36] Srikhaow A., Butburee T., Pon-On W., Srikhirin T., Uraisin K., Suttiponpanit K., Chaveanghong S., Smith S.M. (2020). Efficient Mercury Removal at Ultralow Metal Concentrations by Cysteine Functionalized Carbon-Coated Magnetite. Appl. Sci..

[37] Katok K.V., Whitby R.L.D., Fayon F., Bonnamy S., Mikhalovsky S.V., Cundy A.B. (2013). Synthesis and Application of Hydride Silica Composites for Rapid and Facile Removal of Aqueous Mercury. Chemphyschem.

[38] Liu Y., Wang Z., Huang Y., Zhu S., Yao Y. (2024). Enhanced Hg(II) removal by polyethylenimine-modified fly ash-based tobermorite. Colloids Surfaces A Physicochem. Eng. Asp..

[39] Johari K., Saman N., Mat H. (2014). A comparative evaluation of mercury(II) adsorption equilibrium and kinetics onto silica gel and sulfur-functionalized silica gels adsorbents. Can. J. Chem. Eng..

[40] Bansal M., Ram B., Chauhan G.S., Kaushik A. (2018). L-Cysteine functionalized bagasse cellulose nanofibers for mercury (II) ions adsorption. Int. J. Biol. Macromol..

[41] Thommes M., Cychosz K.A. (2014). Physical adsorption characterization of nanoporous materials: Progress and challenges. Adsorption.

[42] Natusch D.F.S., Porter L.J. (1970). Direct detection of mercury(II)–thio-ether bonding in complexes of methionine and S-methylcysteine by 1H nuclear magnetic resonance. J. Chem. Soc. D Chem. Commun..

[43] Jayalath S., Larsen S.C., Grassian V.H. (2018). Surface adsorption of Nordic aquatic fulvic acid on amine-functionalized and non-functionalized mesoporous silica nanoparticles. Environ. Sci. Nano.

[44] Parker S.F. (2013). Assignment of the vibrational spectrum of l-cysteine. Chem. Phys..

[45] Sebben D., Pendleton P. (2014). Infrared spectrum analysis of the dissociated states of simple amino acids. Spectrochim. Acta Part A Mol. Biomol. Spectrosc..

[46] Fu K.L., Yao M.Y., Wang D.B., Zhao H.C., Cheng G.W., Yang S. (2019). Removal of Hg^2+^ from flue gas by petroleum thioether. IOP Conf. Ser. Earth Environ. Sci..

[47] Kurihara T., Noda Y., Takegoshi K. (2019). Capping Structure of Ligand–Cysteine on CdSe Magic-Sized Clusters. ACS Omega.

[48] Nakamoto K. (1963). Infrared Spectra of Inorganic and Coordination Compounds.

[49] Di Michele A., Diodati P., Morresi A., Sassi P. (2009). Mercury acetate produced by metallic mercury subjected to acoustic cavitation in a solution of acetic acid in water. Ultrason. Sonochem..

[50] Trochimczuk A.W. (1998). Chelating resins with N-substituted diamides of malonic acid as ligands. Eur. Polym. J..

[51] Vickerman J.C., Gilmore I.S., Ian S. (2009). Surface Analysis: The Principal Techniques.

[52] Herrera-Gomez A. (2020). Uncertainties in photoemission peak fitting accounting for the covariance with background parameters. J. Vac. Sci. Technol. A Vac. Surf. Film..

[53] Beamson G., Briggs D. (1993). High Resolution XPS of Organic Polymers: The Scienta ESCA300 Database. J. Chem. Educ..

[54] Pérez O., Odio O.F., Reguera E. (2022). XPS as a probe for the bonding nature in metal acetates. New J. Chem..

[55] Schecher W.D., McAvoy D.C. (1994). MINEQL+: A Chemical Equilibrium Program for Personal Computers.

[56] Lv J., Luo L., Zhang J., Christie P., Zhang S. (2012). Adsorption of mercury on lignin: Combined surface complexation modeling and X-ray absorption spectroscopy studies. Environ. Pollut..

[57] Sing K. (2001). The use of nitrogen adsorption for the characterization of porous materials. Colloids Surf. A Physicochem. Eng. Asp..

[58] Bandosz T.J. (2006). Activated Carbon Surfaces in Environmental Remediation.

[59] Kruk M., Jaroniec M. (2001). Gas Adsorption Characterization of Ordered Organic−Inorganic Nanocomposite Materials. Chem. Mater..

[60] Thielemann J.P., Girgsdies F., Schlögl R., Hess C. (2011). Pore structure and surface area of silica SBA-15: Influence of washing and scale-up. Beilstein J. Nanotechnol..

[61] Baalousha M. (2009). Aggregation and disaggregation of iron oxide nanoparticles: Influence of particle concentration, pH and natural organic matter. Sci. Total. Environ..

[62] Moran-Salazar R.G., Carbajal-Arizaga G.G., Gutierréz-Ortega J.A., Badillo-Camacho J., Manríquez-González R., Shenderovich I.G., Gómez-Salazar S. (2023). As(V) removal from aqueous media using an environmentally friendly zwitterion L-cysteine functionalized silica adsorbent. Chem. Eng. Sci..

[63] Brinker J.C., Scherer G.W. (1990). Sol-Gel Science.

[64] Quirarte-Escalante C.A., Soto V., de la Cruz W., Porras G.R., Manríquez R., Gomez-Salazar S. (2009). Synthesis of Hybrid Adsorbents Combining Sol−Gel Processing and Molecular Imprinting Applied to Lead Removal from Aqueous Streams. Chem. Mater..

[65] Zhang J., Yang Y., Ding J. (2023). Information criteria for model selection. WIREs Comput. Stat..

[66] Akcakayiran D., Mauder D., Hess C., Sievers T.K., Kurth D.G., Shenderovich I., Limbach H.-H., Findenegg G.H. (2008). Carboxylic Acid-Doped SBA-15 Silica as a Host for Metallo-supramolecular Coordination Polymers. J. Phys. Chem. B.

[67] Shenderovich I.G. (2021). Experimentally Established Benchmark Calculations of 31 P NMR Quantities. Chem. Methods.

[68] Gutiérrez-Ortega J.A., Gómez-Salazar S., Shenderovich I.G., Manríquez-González R. (2020). Efficiency and lead uptake mechanism of a phosphonate functionalized mesoporous silica through P/Pb association ratio. Mater. Chem. Phys..

[69] Shenderovich I.G., Mauder D., Akcakayiran D., Buntkowsky G., Limbach H.-H., Findenegg G.H. (2007). NMR Provides Checklist of Generic Properties for Atomic-Scale Models of Periodic Mesoporous Silicas. J. Phys. Chem. B.

[70] Eichele K. (2021). WSolids1-Solid State NMR Simulations User Manual. http://anorganik.uni-tuebingen.de/klaus/soft/wsolids1/wsolids1.pdf.

